# Eye health indicators for universal health coverage: results of a global expert prioritisation process

**DOI:** 10.1136/bjophthalmol-2020-318481

**Published:** 2021-03-12

**Authors:** Ian McCormick, Islay Mactaggart, Serge Resnikoff, Debbie Muirhead, GV Murthy, Juan Carlos Silva, Andrew Bastawrous, Jude Stern, Karl Blanchet, Ningli Wang, Mayinuer Yusufu, Andrew Cooper, Michael Gichangi, Eye Health Indicators Prioritisation Project Group, Matthew J Burton, Jacqueline Ramke

**Affiliations:** 1 International Centre for Eye Health, London School of Hygiene & Tropical Medicine, London, UK; 2 Brien Holden Vision Institute, Sydney, New South Wales, Australia; 3 School of Optometry and Vision Science, University of New South Wales, Sydney, New South Wales, Australia; 4 The Fred Hollows Foundation Melbourne, Melbourne, Victoria, Australia; 5 Indian Institutes of Public Health, Hyderabad, India; 6 Pan American Health Organization, Bogota, Colombia; 7 International Agency for the Prevention of Blindness, London, UK; 8 Faculty of Medicine, University of Geneva, Geneva, Switzerland; 9 Beijing Institute of Ophthalmology, Beijing Tongren Eye Center, Beijing Tongren Hospital, Capital Medical University, Beijing, China; 10 Vision Catalyst Fund, London, UK; 11 Ophthalmic Services Unit, Kenya Ministry of Health, Nairobi, Kenya; 12 Moorfields Eye Hospital, London, UK; 13 School of Optometry and Vision Science, The University of Auckland, Auckland, New Zealand

**Keywords:** public health

## Abstract

**Introduction:**

In its recent *World Report on Vision*, the WHO called for an updated approach to monitor eye health as part of universal health coverage (UHC). This project sought to develop a consensus among eye health experts from all world regions to produce a menu of indicators for countries to monitor eye health within UHC.

**Methods:**

We reviewed the literature to create a long-list of indicators aligned to the conceptual framework for monitoring outlined in WHO’s *World Report on Vision*. We recruited a panel of 72 global eye health experts (40% women) to participate in a two-round, online prioritisation exercise. Two-hundred indicators were presented in Round 1 and participants prioritised each on a 4-point Likert scale. The highest-ranked 95 were presented in Round 2 and were (1) scored against four criteria (feasible, actionable, reliable and internationally comparable) and (2) ranked according to their suitability as a ‘core’ indicator for collection by all countries. The top 30 indicators ranked by these two parameters were then used as the basis for the steering group to develop a final menu.

**Results:**

The menu consists of 22 indicators, including 7 core indicators, that represent important concepts in eye health for 2020 and beyond, and are considered feasible, actionable, reliable and internationally comparable.

**Conclusion:**

We believe this list can inform the development of new national eye health monitoring frameworks, monitor progress on key challenges to eye health and be considered in broader UHC monitoring indices at national and international levels.

## Introduction

In its first *World Report on Vision* released in 2019, WHO included the strengthening of health information systems (HIS) among its five global priority areas for action.^1^ This recognises the critical role of HIS to provide information—from population-based surveys, facility-based sources and administrative data—to guide health policy, management and clinical care. Among WHO’s recommended actions were to strengthen national capacity to collect, analyse and use data on eye health, and the creation of a global indicator menu for eye health from which countries can select relevant indicators.^1^

The priority placed on HIS in the *World Report on Vision* also reflects the limited progress made to date. Several lists of indicators have accompanied global eye health initiatives over the past two decades.^2–5^ Inconsistent reporting against these lists over time may be due to under-investment in district-level HIS capacity in low-income and middle-income settings, the vertical nature of many eye health systems, variable levels of engagement from national eye care planners and limited public–private sector cooperation.^6–8^ In addition, a lack of policy imperative may be due to an absence of eye health indicators in WHO’s global health monitoring frameworks to date.^9 10^

Based on these and other challenges, in its *Universal Eye Health: A Global Action Plan 2014–2019* 4 (hereafter *‘*GAP*’*) in 2013, WHO emphasised the need for eye care to be integrated into broader health planning. The *World Report on Vision* went further to state that eye health should be considered an essential component of universal health coverage (UHC).^1^ Monitoring global eye health as part of UHC and the United Nations’ Sustainable Development Goals^11^ requires an updated menu of indicators aligned with the UHC dimensions of access, quality, financial risk protection and equity.

Here we report a collaborative prioritisation process to generate a menu of indicators that may be used by governments to monitor and improve eye health and eye health services at the national level, and to support progress towards achieving UHC. This work was undertaken as part of the *Lancet Global Health Commission on Global Eye Health*.^12^

## Methods

### Study design

A two-round, prioritisation exercise was undertaken between February and April 2020 using an online survey platform (www.qualtrics.com). All panellists’ responses were de-identified throughout, however, individuals were provided the option to join a study authorship group.

### Participants

A project steering group (the co-authors) was convened to guide the development of the initial long-list of indicators, nominate panellists from a network of global eye health experts, review indicator scoring and develop the final menu. We aimed to recruit panellists from all Global Burden of Disease (GBD) Super Regions,^13^ with equal numbers of men and women per region. In total 74 out of 84 invited panellists participated in Round 1 and 72 went on to complete Round 2 (response rate after Round 2, 85.7%). Men were 59.7% of the Round 2 panel, similar to the proportion among all invitees. Eleven members of the steering group participated, five from a ‘global’ (non-Regional) perspective. Thirty-nine countries and all GBD Super Regions had participants in both rounds and 85% of the Round 2 panel represented low-income or middle-income countries (table 1). Round 2 panellists most frequently reported their roles within eye health as ‘management/leadership’ (25.0%), ‘epidemiology’ (12.5%), ‘clinician/practitioner’ (12.5%), ‘eye health services research’ (9.7%), ‘government/Ministry of Health’, ‘clinical research’ and ‘international institution’ (all 6.9%).

**Table 1:** Round 2 response rate among invitees by Global Burden of Disease (GBD) Super Region and sex

GBD Super Region	Female			Male			Total		
	Completed	Invited	Response rate	Completed	Invited	Response rate	Completed	Invited	Response rate
	N	N	%	N	N	%	N	N	%
Sub-Saharan Africa	4	5	80.0	12	13	92.3	16	18	88.9
South East Asia, East Asia and Oceania	7	9	77.8	4	6	66.7	11	15	73.3
Latin America and Caribbean	6	8	75.0	6	6	100.0	12	14	85.7
South Asia	3	3	100.0	9	10	90.0	12	13	92.3
North Africa and Middle East	1	2	50.0	5	6	83.3	6	8	75.0
High Income	3	3	100.0	3	4	75.0	6	7	85.7
Central Europe, Eastern Europe and Central Asia	2	2	100.0	2	2	100.0	4	4	100.0
‘Global perspective’	3	3	100.0	2	2	100.0	5	5	100.0
Total	29	35	82.6	43	49	87.8	72	84	85.7

### Initial indicator selection

**Figure 1:**
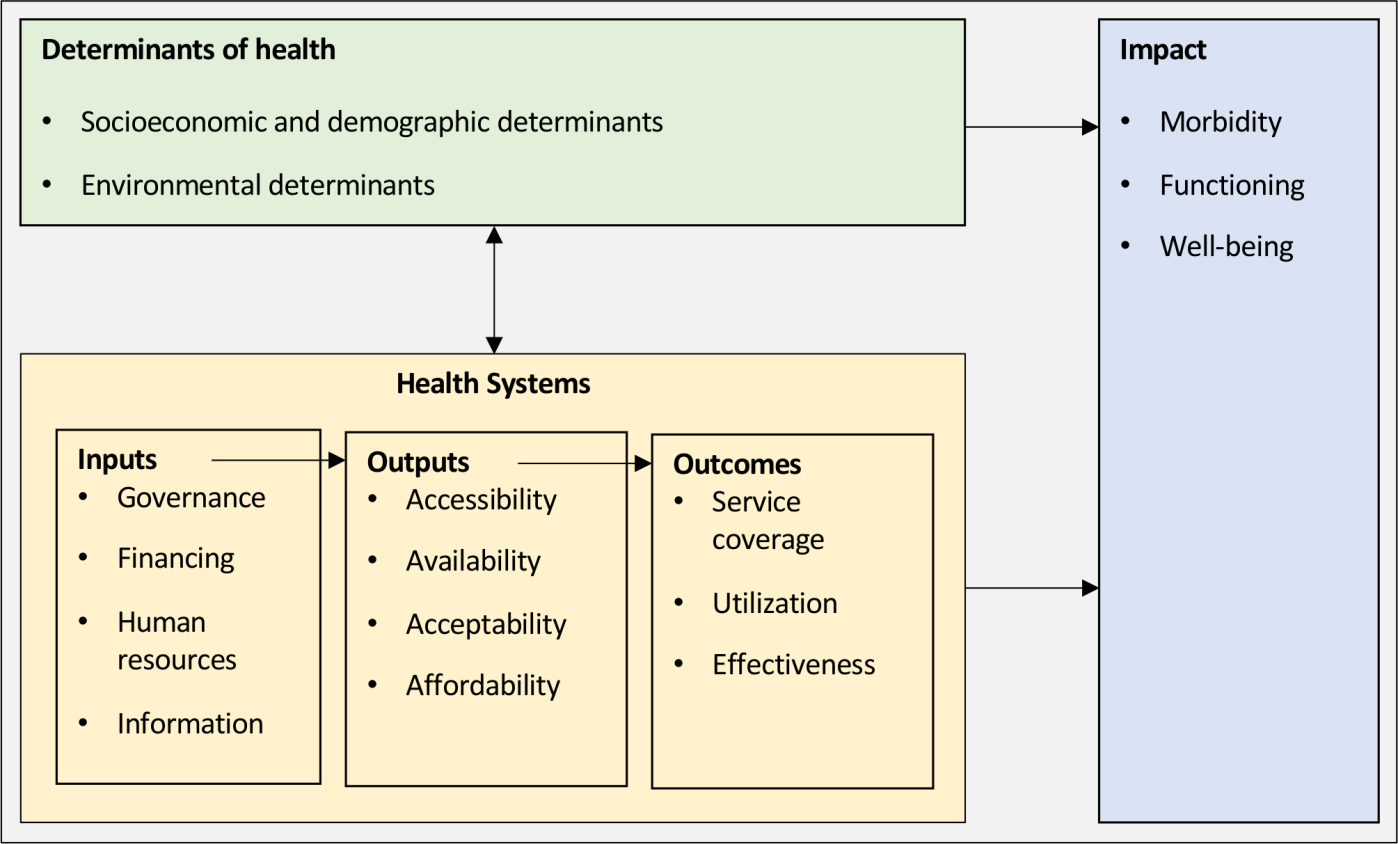
Domains of measurement of health information systems (reproduced from the *World Report on Vision*).

A long-list of indicators was compiled with reference to previously proposed eye health indicators and existing international health and health systems indicator lists, adapted for relevance to the eye health sector where necessary. This long-list was mapped to the domains of measurement of HIS used in the *World Report on Vision* (adapted from the 2012 WHO *Framework and standards for country health information systems*) (figure 1). When panellists were invited to participate, they were asked to suggest additional indicators for consideration. The steering group reviewed all indicators identified, only excluding obvious duplicates in order to avoid biassing the pool of potential indicators. At the end of this process 200 indicators were included (online supplemental appendix 1).

10.1136/bjophthalmol-2020-318481.supp1 Supplementary data

### The prioritisation exercise: round 1

Panellists scored the indicators based on perceived priority in their context. Priority was scored from 1 to 4 on a Likert scale, with 1 representing the lowest priority ('no need to collect') and 4 the highest priority ('essential to collect') (online supplemental appendices 2 and 3). A fifth option, (0 = ‘redundant’) was included to allow for the fact that the long-list had not been heavily edited and some overlap of indicator concepts was possible. A *priority score* for each indicator was calculated by summing the products of two dimensions: the Likert scale score (1–4) x the number times each indicator received that score.

10.1136/bjophthalmol-2020-318481.supp2 Supplementary data

At the end of Round 1, an initial threshold for continued inclusion was set at or above the median score. Indicators scoring in the top half were merged where there was sufficient overlap in concepts to do so. Indicators not scoring in the top half were reviewed to determine if any concepts deemed essential to score in Round 2 had been omitted and should be included to ensure representation (online supplemental appendices 2 and 3). In total, 95 indicators were forwarded to Round 2.

### The prioritisation exercise: round 2

Each of the 95 indicators were scored against four new criteria. The panel were asked to indicate their agreement on a 4-point Likert scale (1 = ‘strongly disagree’, 2 = ‘disagree’, 3 = ‘agree’, 4 = ‘strongly agree’) as to whether each indicator was feasible, actionable, reliable and internationally comparable (table 2). In addition, the panel selected 10 indicators they considered to be ‘core’ indicators, described as those which all countries could be encouraged to adopt. These were ranked 1 (most important) to 10.

**Table 2:** Criteria used to score Round 2 indicators

Criteria	Definition
Feasible	The indicator can be derived using either available data (eg, routine monitoring) or purposeful data collection (eg, population-based survey, clinic-based study) without substantial additional resources
Actionable	The indicator measures an aspect of eye health within health systems that may be used at a national level to create change through policymaking or strategy development
Reliable	The indicator returns similar results when measuring a stable phenomenon (eg, measurement has a sufficient degree of objectivity)
Internationally comparable	Reporting countries can comply with the relevant data definition; any differences in the indicator values between countries reflect issues in health systems rather than differences in data collection methodologies, coding or measurements

Scores were calculated in the same way as Round 1. Each indicator was scored on the criteria separately and a *composite score* of all four was calculated, with all criteria weighted equally. Each indicator was assigned a rank position from 1 to 95 for each of the four criterion and the overall composite score. The ranking of indicators 1 to 10 as core indicators was calculated in a similar way: a vote for first place awarded 10 points, second place awarded 9 points and so on. Points were multiplied by the number of times an indicator received that vote position for an overall *core score* (online supplemental appendices 2 and 3). A ranking of 1 to 95 was given based on this scoring and this ranking was used in all subsequent analysis. Indicators with the same score were ranked equal.

We arrived at a list of 30 priority eye health indicators by ranking the Round 2 selections using two metrics:

1. The rank of the indicator based on the *core score.*
2. The rank of the indicator based on the *composite score*

We plotted the *core* and *composite* scores against each other and selected the 30 indicators that scored most highly by both ranking methods, by expanding the ‘gating’ equally along both axes until the selected area included 30 indicators (online supplemental appendices 2 and 3). The selected indicators, therefore, scored relatively highly for both.

### Development of the indicator menu

Starting with the top 30 indicators from Round 2, we developed the detailed indicator menu presented in box 1. In this step we aimed to:

- Ensure alignment with UHC dimensions of access, quality, financial protection and equity
- Avoid repetition or misclassification of themes within and across domains
- Avoid over-representation or under-representation of domains
- Identify any omissions related to the five most prevalent causes of vision impairment globally (cataract, uncorrected refractive error, glaucoma, age-related macular degeneration, diabetic retinopathy).^14^

Box 1 **Consolidated indicators menu, integrating global panel indicator preferences with a conceptual framework for monitoring eye health as part of universal health coverage.**

**Equity statement**

All indicators summarising population-based and eye care facility-based data should report metrics disaggregated by key equitydimensions of sex, place of residence (PoR), socioeconomic position (SEP) and disability status, where available. Additional options, suchethnicity or marital status, can be recorded by countries as appropriate.

**Inputs and processes**

**Governance**

**G1** Eye health is integrated into the national health strategy/plan (or the relevant specific plan, for example, non-communicable diseases)

 ► G1.1 National health plan includes human resources for eye care (Y/N)

 ► G1.2 Eye health is integrated into the plans, policies and budget of other initiatives such as:

  – G1.2.1 National essential package of health services (Y/N)

  – G1.2.2 Primary healthcare (Y/N)

  – G1.2.3 Maternal and child healthcare (Y/N)

  – G1.2.4 Diabetes care (Y/N)

  – G1.2.5 School health programmes (Y/N)

  – G1.2.6 Healthy ageing programmes (Y/N)

 ► G1.3 National eye health policies, plans and programmes refer to a multisectoral approach/engagement with other sectors (Y/N)

  – *If a national eye health strategy/ plan is unavailable or not up-to-date, record as N*

**G2** Is the national eye health plan informed by recent evidence (Y/N):

 ► G2.1 Time since cited population-based data was collected (in months/years)

 ► G2.2 Time since cited Eye Care Service Assessment Tool (ECSAT) data was collected (in months/years)

**Finance**

**F1** Eye health is integrated into the national health budget (Y/N)

 – *Requires a working group to develop sub-indicators and metadata*

**F2** Eye health is included in national health finance pooling mechanism (Y/N)

 – *Scaled response based on scoring outcomes of sub-indicators in ‘checklist’*

   If yes, the range/number/list of services addressing leading causes of vision impairment (VI) included:

 ► F2.1 Outpatient consultation (Full/Partial/No)

 ► F2.2 Cataract (Full/Partial/No)

 ► F2.3 Refraction services (Full/Partial/No)

 ► F2.4 Glaucoma medication/surgery (Full/Partial/No)

 ► F2.5 Diabetic retinopathy – laser/anti-vascular endothelial growth factor (VEGF) (Full/Partial/No)

**F3** Proportion of population covered via national health finance pooling mechanisms that includes eye care services:

 ► F3.1 Proportion covered for: Outpatient consultation

 ► F3.2 Proportion covered for: Cataract

 ► F3.3 Proportion covered for: Refraction services

 ► F3.4 Proportion covered for: Glaucoma medication/surgery

 ► F3.5 Proportion covered for: Diabetic retinopathy – laser/anti-VEGF

**Infrastructure**

**I1** Eye health facility density and distribution, disaggregated by:

 ► I1.1 Primary

 ► I1.2 Secondary

 ► I1.3 Tertiary

 ► I1.4 Low vision services

  – *By PoR (urban/rural), total numbers (public and private) per million population*

  – *Additional subnational administrative or geographical divisions as relevant to setting*

 – *Additional dimension: Access to primary eye care and cataract surgery via global positioning system data and geospatial modelling*

**I2** Percentage of neonatal units providing screening for retinopathy of prematurity nationally

**Supply chain**

**SC1** Pharmaceuticals specifically for eye care on the National Essential Medicines List

*  – Total number and proportion compared with a normative standard for eye health pharmaceuticals (eg, WHO or International Agency for the Prevention of Blindness list)*

**Information**

**INFO1** Existence of a National Health Information System that includes eye care service data (Y/N)

**Eye health workforce**

**HR1** Eye health worker density and distribution, disaggregated by:

 ► HR1.1 Ophthalmologist

 ► HR1.2 Optometrist

 ► HR1.3 Ophthalmic nurse

 ► HR1.4 Other allied ophthalmic personnel (as relevant to country)

  – *By PoR (urban/rural), total number per million population, and by age groups and sex*

  – *Additional subnational administrative or geographical divisions as relevant to setting*

  – *Additional dimension: 5-year trends per cadre*

**HR2** Is Primary Eye Care integrated into the national Primary Healthcare training (if applicable)? (Y/N)

**Outputs**

**Access**

**AC1** Cataract surgical rate

 – *Total number per million population and including variation in rate across urban/rural or districts*

 – *Additional dimension: 5-year trend in cataract surgical rate*

 – *Additional dimension: Surgical case-mix in terms of preoperative visual acuity*

**Quality and safety**

**Q1** Cataract surgical outcome (visual acuity)

 – *Proportion of eyes with a 'good' outcome (6/18 or better)*

 – *Proportion of eyes with a ‘poor’ outcome (worse than 6/60)*

**Q2** Number of priority eye conditions with quality of care/clinical practice guidelines endorsed by relevant regulatory bodies

 ► Q2.1 Cataract (Y/N)

 ► Q2.2 Refractive error (Y/N)

 ► Q2.3 Glaucoma (Y/N)

 ► Q2.4 Age-related macular degeneration (Y/N)

 ► Q2.5 Diabetic retinopathy (Y/N)

 ► Q2.6 Child eye health (Y/N)

**Responsiveness/affordability**

**AF1** Median (range) of out-of-pocket payment made for cataract surgery as a proportion of median monthly household (or individual)income

 – *Report median and mean payment made at point of service (excluding transport, accommodation, sustenance)*

 – *Disaggregated by provider type (government/public, private for profit, private non-governmental organisation/charity)*

 – *Additional dimension: proportion reported for poorest vs wealthiest quintiles*

**Outcomes**

**Coverage**

**C1** Cataract surgical coverage and effective cataract surgical coverage

 – *CSC (cataract surgical coverage), eCSC (effective CSC), ‘quality gap’ reported, disaggregated by age, sex, SEP, PoR as available*

**C2** Refractive error coverage and effective refractive error coverage

 – *REC (refractive error coverage), eREC (effective REC), ‘quality gap’ reported, disaggregated by age, sex, SEP, PoR as available*

**C3** Coverage of diabetic retinopathy screening of all people with diabetes (at the frequency recommended in national guidelines)

 – *Requires a working group to develop complete indicator metadata*

 – *Disaggregated by age, sex, SEP, PoR as available*

**C4** Coverage of school eye health programmes for schools nationally

 – *Proportion of schools receiving screening in the past 12 months*

 – *Disaggregated by primary and secondary schools*

**Impact**

**Improved outcomes**

**P1** Prevalence of VI

 ► P1.1 Distance VI prevalence, by WHO categories

 ► P1.2 Near VI prevalence, by WHO definition

  – *From population-based surveys, disaggregated by age, sex, SEP, PoR as available*

**P2** Cause-specific prevalence of VI

 – *Prevalence of vision-impairing priority eye conditions from population-based surveys, disaggregated by age, sex, SEP, PoR as available*

 ► P2.1 Avoidable blindness/severe VI/moderate VI/mild VI prevalence disaggregated by age, sex, SEP, PoR as available

 – *Aggregated from VI causes assigned in surveys*

**P3** Prevalence of childhood VI and blindness

  – *Blindness/severe VI/moderate VI/mild VI from population-based or key-informant surveys, disaggregated by age, sex, SEP, PoR as available*

**Figure 2:**
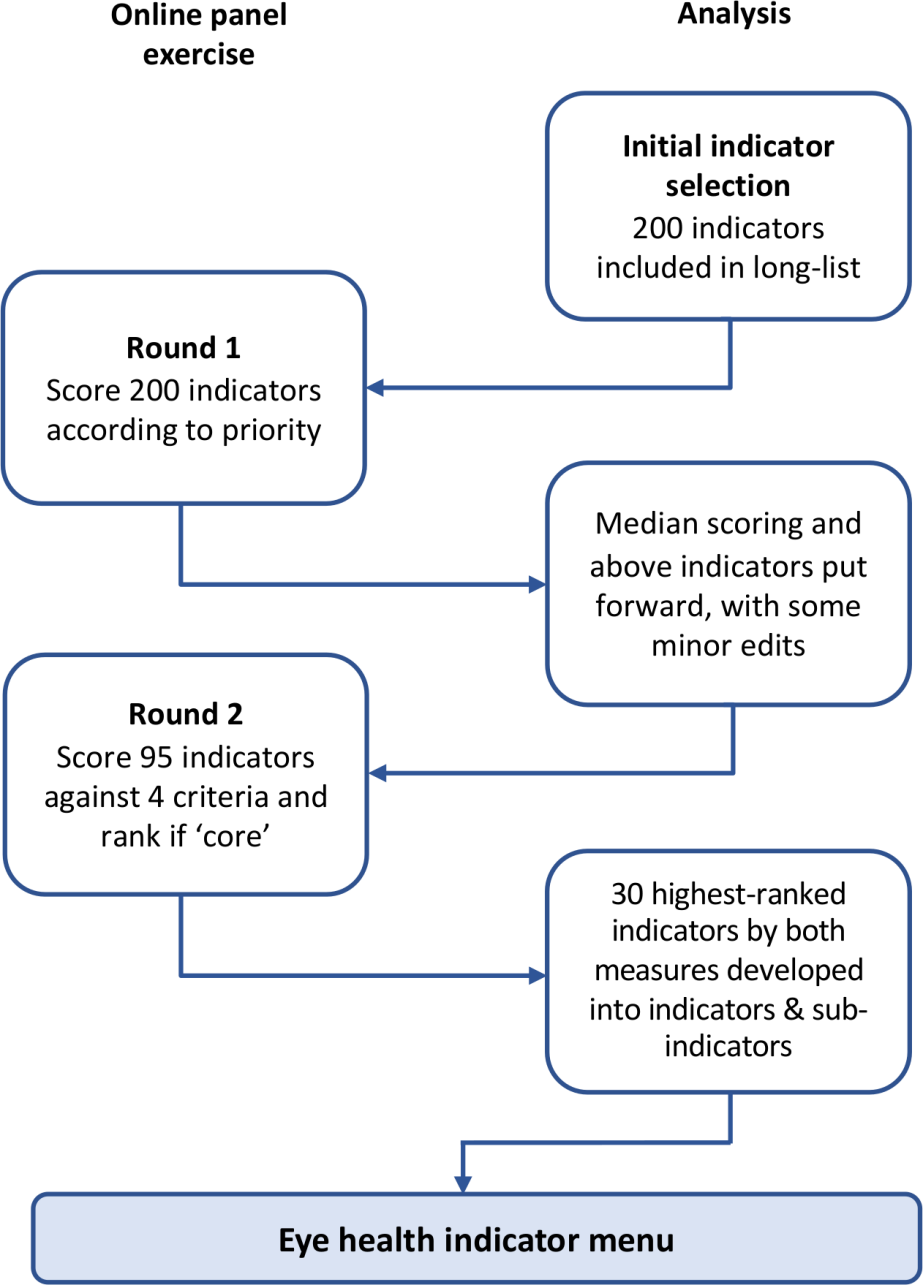
Flowchart describing the process undertaken to develop the eye health indicator menu.

No major edits to key concepts were undertaken. This process is summarised in figure 2.

## Results

Twenty-two distinct eye health indicator concepts were identified (box 1). In compiling the menu, we articulated broader concepts by specifying 39 ‘sub-indicators’ (see bullet points under indicator titles). It is anticipated that these could be used in support of defining the broader indicators, for example, whether or not eye health is integrated into national health planning. Sub-indicators for the concept of eye health financing integration are not yet developed and, once included, will increase the scope of the menu in this domain.

The steering group selected seven core indicators for monitoring eye health as part of countries’ progress towards UHC. These are set out in table 3.

**Table 3:** Core indicators to monitor universal access to quality, affordable eye care services when needed

Indicator	Definition	Rationale	Data sources	Responsible entity	Comments
**Accessibility of eye health services**					
Eye health facility density and distribution	By place of residence (urban/rural), total numbers (public and private) of primary, secondary, tertiary and low vision services per million population Additional subnational administrative or geographical divisions as relevant to setting	Place of residence should not be a barrier to accessing eye health services	Facility records, population data	Health ministry	Informs policy and planning about location of eye health services in relation to population density. Outreach programmes may be planned according to gaps in geographical access to static services
Eye health worker density and distribution	By place of residence (urban/rural), total numbers of ophthalmologist, optometrist, ophthalmic nurses and other allied ophthalmic personnel per million population Additional subnational administrative or geographical divisions as relevant to setting	Availability and accessibility of eye health workers dictates access to care	Facility records, data from professional or regulatory bodies, population data	Health ministry	Informs policy and planning on recruitment and distribution of human resources for eye health. Known disparities exist in the number and distribution of trained eye care personnel between countries and by urban and rural settings within countries
**Affordability of eye health services**					
Coverage of national health finance pooling mechanisms that include eye care services	Proportion of population covered with health finance pooling mechanisms that include eye care services (considered individually): Outpatient care Cataract Refractive error services Glaucoma treatment Diabetic retinopathy treatment	Cost should not be a barrier to accessing eye care. Proxy for WHO/World Bank UHC financial risk protection indicators; catastrophic and/or impoverishing OOP payments unlikely to be discriminatory for monitoring affordability of elective eye care services	Health finance scheme reports and questionnaires	Health ministry	Informs policy about eye health financing and affordability. Coverage within the lowest wealth quintile should be reported alongside the total population to monitor equitable coverage of eye health financing
OOP payments for cataract surgery	Median (and range) of OOP payment made for cataract surgery as a proportion of median monthly household (or individual) income	Cost should not be a barrier to accessing eye care. Proxy for WHO/World Bank UHC financial risk protection indicators; catastrophic and/or impoverishing OOP payments unlikely to be discriminatory for monitoring affordability of elective eye care services	Population-based surveys	Health ministry (Surveys may be commissioned in collaboration with other stakeholders)	Informs policy about eye health financing and affordability. Additional services could be monitored in the same way
**Effective coverage of cataract and refractive error services**					
Effective cataract surgical coverage	Among the population aged 50 years and older, people with operated cataract and good postoperative presenting visual acuity as a proportion of all people with operated cataract or operable cataract Disaggregated by sex	Sex-disaggregated effective coverage measures the UHC dimensions of access, quality and equity for the leading cause of blindness globally	Population-based surveys	Health ministry (Surveys may be commissioned in collaboration with other stakeholders)	Informs policy and planning about the met and unmet need for cataract surgical services; candidate WHO UHC tracer indicator
Effective refractive error coverage	Adults with refractive error corrected to a pre-defined visual acuity threshold with habitual correction as a proportion of all people with corrected and uncorrected refractive error Disaggregated by sex	Sex-disaggregated effective coverage measures the UHC dimensions of access, quality and equity for the leading cause of vision impairment globally	Population-based surveys	Health ministry (Surveys may be commissioned in collaboration with other stakeholders)	Informs policy and planning about the met and unmet need for refractive error services; candidate WHO UHC tracer indicator
**Prevalence of vision impairment**					
Prevalence of VI	The prevalence of all cause distance and near VI (according to WHO definitions) Disaggregation by key equity measures Disaggregation by avoidable vs non-avoidable	Proxy measure of eye health; a measure of programmatic success in journey towards eye health as part of UHC	Population-based surveys	Health ministry (Surveys may be commissioned in collaboration with other stakeholders)	Disaggregated VI prevalence estimates inform policy makers about the impact of eye health systems on eye health among population subgroups

OOP, out-of-pocket; UHC, universal health coverage; VI, vision impairment.

## Discussion

This process engaged a large panel of global eye health experts representing all GBD Super Regions and developed a quantitative approach to prioritise existing indicators. The steering group refined the highest ranked selections to produce a menu of indicators for governments to monitor and improve eye health and eye health services, aligned with UHC and the Sustainable Development Goal on health and in keeping with WHO’s call for such a menu in the *World Report on Vision*. We believe the core indicators highlighted here, if collected by all countries, could allow governments, and supranational organisations, to track progress on key challenges within eye health and UHC. Otherwise, the menu is not intended to be prescriptive; countries could select indicators according to priorities based on population need. We recognise that some countries will likely benefit from collecting and reporting fewer, more important eye health indicators as accurately as possible.

The core indicators include two candidate WHO UHC service coverage indicators: effective cataract surgical coverage (eCSC) and effective refractive error coverage (eREC).^15 16^ Effective coverage has been acknowledged as a useful measure of progress towards UHC as it includes dimensions of quality, access and, where disaggregated, equity. Both eCSC and eREC were omitted from a recent global UHC analysis because of limited data availability,^17^ an issue which must be addressed.

The standard UHC financial risk protection indicators (catastrophic and impoverishing expenditure) adapted to eye health scored lowest among the 95 indicators in Round 2. This likely reflects anticipated complexities in data collection and the possibility that, for non-emergency healthcare, they may not be sufficiently discriminatory. Instead, we have proposed two new proxy measures for financial risk protection. These are not intended as direct replacements for catastrophic and impoverishing expenditure indicators, rather what might be achievable within the constraints of eye health data availability. They will require additional work to develop a full metadata description but provide a way to track eye health insurance coverage (for multiple conditions) and out-of-pocket (OOP) payments for treatment of cataract, the most common cause of blindness globally. The WHO has acknowledged that monitoring the intersection between service coverage and OOP expenditure is key to assessing progress towards UHC.^18^ There may be value in expanding this OOP payment indicator to include refractive error correction and a pilot test for one or both of these will be undertaken in the near future within the Rapid Assessment of Avoidable Blindness (RAAB), a well-established population-based survey method.^19^ We acknowledge that relying on OOP payment data obscures those people who do not present to care due to unaffordable cost, an essential group to identify and reach for UHC to be realised.^20^

Place of residence should not be a barrier to accessing care, however, human resources for eye care are skewed towards urban settings.^21 22^ We have included two provider-side measures of access: human resources and infrastructure for eye care per capita. These can be reported more frequently than service coverage estimates and at little cost. The density of eye health workers (by cadre) features in all previous eye health indicator lists. In order to be UHC-aligned, countries must expand beyond aggregate numbers of personnel, and monitor their distribution by rural/urban and public/private settings. Disaggregation should also be applied to the monitoring of eye care facility density and distribution. This metric could be enhanced by geocoding infrastructure and integrating geographical coverage with other population data to apply a spatial component to eye health planning.^23^

The final concept represented in the core indicators selection is the impact of countries’ eye health systems on population health in terms of the prevalence of vision impairment. Vision impairment affects individuals’ quality of life but is also a broader development issue affecting education and employment.^24^ Vision impairment prevalence has been the key measure of eye health for decades and continues to be useful at the national level for monitoring and planning and at the global level for advocacy.

Beyond the core indicators, key concepts represented in the menu include the integration of eye health into national health planning and financing. We acknowledge that defining and measuring the degree of integration in these areas requires further discussion, particularly for health financing. The integration of primary eye care into primary healthcare planning, financing and training programmes is also included, along with integration of eye health into HIS.

The importance of data disaggregation to monitor eye care equity across the menu should not be understated. We have included an equity statement for consideration across the list and believe equity-relevant monitoring is essential to ensure the most gains are made among population groups with the most need.

Our menu has substantial overlap with WHO’s *GAP* indicator list, including its six key indicators on vision impairment, human resources for eye care and cataract surgical services. New concepts in our list include eye care insurance coverage and affordability, the UHC dimensions of quality and equity, eye health infrastructure and information systems, primary eye care, child eye health, refractive error and diabetic retinopathy. Our proposed indicators are not only UHC-aligned but also address many areas of global eye health prioritised in a recent ‘grand challenges’ global Delphi process.^12^

There are some notable absences from the final menu. The concept of ‘people-centred’ eye care proposed in the *World Report on Vision* is not represented. We presented 13 ‘responsiveness’ indicators in Round 1, including 7 patient-reported outcome indicators, but the panel prioritised none; this will require further study. Disease-specific indicators for glaucoma and age-related macular degeneration were potentially under-represented in the initial long-list and not prioritised by the panel, despite their prominence as causes of vision loss globally.^14^ This may be because the natural history of these conditions make monitoring more complex than for cataract or refractive error. Appropriate coverage indicators for these conditions will require further investigation, and as the menu evolves, more ‘difficult-to-measure’ concepts would ideally be included.^12^ Trachoma and onchocerciasis were not prioritised, likely reflecting the progress made in these areas in recent years. However, we expect endemic countries would continue to report against indicators aligned with their elimination programmes. Unilateral vision impairment, associated with, for example, infectious corneal ulcers, is not included in the menu but was identified as a knowledge gap in the *World Report on Vision* and could be included in future as it gains priority in eye health planning. The *GAP* indicator for evidence of research on the cost-effectiveness of eye health programmes was not prioritised, but more evidence of cost-effectiveness may strengthen the case for resource allocation. Finally, broader health and financing indicators potentially relevant to eye health (demographics, non-communicable diseases, water and sanitation, government health spending) were not prioritised but could be obtained from other national reporting mechanisms to support eye care planning as appropriate.

We recognise that generating this list is insufficient in isolation, and several challenges must be addressed for these indicators to be successfully integrated into countries’ HIS and monitoring frameworks. Fortunately, the priority given to HIS in the *World Report on Vision* and the potential inclusion of eCSC and eREC in the next list of WHO UHC indicators provides impetus for action. In addition, countries will benefit from the ongoing refinement of tools such as WHO’s Eye Care Services Assessment Tool^25^ and RAAB and its Planning Module.^19^ These tools strengthen national HIS capacity by providing guidance on data collection and interpretation for a range of indicators included in our list. Several of the new indicators proposed here require indicator metadata which would ideally be generated by subject-specific expert working groups working collaboratively with countries. Alongside indicator development, appropriate target-setting also requires consultation. Further, there are financial and logistical challenges for countries to routinely collect national-level population health data, so rapid surveys of vision impairment and eye care services have often been carried out at the subnational level to aid local planning. In the absence of increased national-level data collection, modelled estimates will be required to provide data for global estimates and regional and national comparisons with any degree of regularity.

We propose that new indicators in this menu be field-tested in several contrasting settings, and that the menu be regularly reviewed and updated according to user feedback. Such reviews would ideally assess whether data collection and indicator usage are viable and valuable for both national and subnational planning, as well as for generating global eye health estimates. These steps require ongoing engagement and resourcing to develop and maintain the utility of the menu. This may be encouraged by a centralised eye health data repository.

### Limitations

This study has several limitations. First, inherent in a study that recruits experts, the indicators prioritised reflect the preferences of those invited to participate. We aimed to be as geographically representative as possible, however, the North Africa and Middle East, High Income and Central Europe, Eastern Europe and Central Asia Super Regions had few panellists. Further, despite aiming for gender parity, only 40% of the panel were women. A more diverse panel may have generated a different set of indicators. Second, the online exercise was only available in English, however, no nominated panel members were unable to participate due to language constraints. Third, personal interests and familiarity with some concepts over others may have led to confirmation bias in scoring by panel members. The overlap with existing GAP indicators may be a reflection of this, however, the menu does include many new concepts. Finally, detailed explanations of new concepts are required which was beyond the scope of this prioritisation project.

## Conclusion

This process sought a broad consensus from 72 eye health experts from all world regions to produce a menu of indicators for countries to monitor eye health as part of UHC. From a long-list of 200, the final menu consists of 22 indicators that represent important concepts in eye health for 2020 and beyond, and are relatively feasible, actionable, reliable and internationally comparable. The new direction in global eye health set by the *World Report on Vision* must be supported with investment in HIS that include eye health data collection and data monitoring via internationally acceptable indicators. We believe this list is well-placed to inform the development of new national eye health monitoring frameworks and shows where eye health metrics might be incorporated into broader UHC monitoring indices at national and international levels.

## Data Availability

Data are available from the corresponding author upon reasonable request.

## References

[1] World Health Organization . World report on vision. Geneva; 2019.

[2] World Health Organization . A framework and indicators for monitoring vision 2020 – the right to sight: the global initiative for the elimination of avoidable blindness, 2003. Available: https://apps.who.int/iris/bitstream/handle/10665/68600/WHO_PBL_03.92.pdf?sequence=1

[3] World Health Organization . Global initiative for the elimination of avoidable blindness: action plan 2006-2011, 2007. Available: https://www.iapb.org/wp-content/uploads/VISION-2020-Action-Plan-2006-2011.pdf [Accessed 05 Aug 2020].

[4] World Health Organization . Universal eye health: a global action plan 2014-2019, 2013. Available: https://www.who.int/blindness/AP2014_19_English.pdf [Accessed 21.08 2019].

[5] World Health Organization . Catalogue of key eye health indicators in the African region, 2017. Available: https://www.afro.who.int/publications/catalogue-key-eye-health-indicators-african-region [Accessed 26 Nov 2019].

[6] Maïga A , Jiwani SS , Mutua MK , et al . Generating statistics from health facility data: the state of routine health information systems in eastern and southern Africa. BMJ Glob Health 2019;4:e001849. 10.1136/bmjgh-2019-001849 PMC6768347 31637032

[7] Ramke J , Zwi AB , Silva JC , et al . Evidence for national universal eye health plans. Bull World Health Organ 2018;96:695–704. 10.2471/BLT.18.213686 30455517 PMC6238994

[8] Eckert KA , Lansingh VC , McLeod-Omawale J , et al . Field testing project to pilot World Health organization eye health indicators in Latin America. Ophthalmic Epidemiol 2018;25:91–104. 2017/09/26.. 10.1080/09286586.2017.1359848 28945466

[9] World Health Organization . 2018 global reference list of 100 core health indicators (plus health-related SDGs), 2018. Available: https://apps.who.int/iris/bitstream/handle/10665/259951/WHO-HIS-IER-GPM-2018.1-eng.pdf?sequence=1

[10] World Health Organization and International Bank for Reconstruction and Development / The World Bank . Tracking universal health coverage: 2017 global monitoring report.; 2017. Licence: CC BY-NC-SA 3.0 IGO.

[11] United Nations . Transforming our world: the 2030 agenda for sustainable development, 2015. Available: https://sustainabledevelopment.un.org/post2015/transformingourworld

[12] Burton M , Ramke J , Marques A . Lancet global health Commission on global eye health: vision beyond 2020. Lancet Global Health. In Press 2020.

[13] Institute for Health Metrics and Evaluation . Global burden of disease (GBD): about GDB: frequently asked questions, 2020. Available: http://www.healthdata.org/gbd/faq#What%20is%20GBD%202010%20and%20why%20is%20it%20important

[14] Adelson JD , Bourne RRA , Briant PS . Causes of blindness and vision impairment in 2020 and trends over 30 years: evaluating the prevalence of avoidable blindness in relation to “VISION 2020: the Right to Sight”. Lancet Global Health. In Press 2020. 10.1016/S2214-109X(20)30489-7 PMC7820391 33275949

[15] Ramke J , Gilbert CE , Lee AC , et al . Effective cataract surgical coverage: an indicator for measuring quality-of-care in the context of universal health coverage. PLoS One 2017;12:e0172342. 10.1371/journal.pone.0172342 28249047 PMC5382971

[16] McCormick I , Mactaggart I , Bastawrous A , et al . Effective refractive error coverage: an eye health indicator to measure progress towards universal health coverage. Ophthalmic Physiol Opt 2020;40:1–5. 2019/12/28. 10.1111/opo.12662 31879992 PMC7004023

[17] Lozano R , Fullman N , Mumford JE , et al . Measuring universal health coverage based on an index of effective coverage of health services in 204 countries and territories, 1990–2019: a systematic analysis for the global burden of disease study 2019. The Lancet 2020;396:1250–84. 10.1016/S0140-6736(20)30750-9 PMC7562819 32861314

[18] World Health Organization . Thirteenth General programme of work (GPW13) methods for impact measurement, 2020. Available: https://www.who.int/publications/m/item/thirteenth-general-programme-of-work-(gpw13)-methods-for-impact-measurement [Accessed 25 Aug 2020].

[19] Mactaggart I , Wallace S , Ramke J , et al . Rapid assessment of avoidable blindness for health service planning. Bull World Health Organ 2018;96:726–8. 10.2471/BLT.18.217794 30455521 PMC6239001

[20] Ataguba JE , Ingabire M-G . Universal health coverage: assessing service coverage and financial protection for all. Am J Public Health 2016;106:1780–1. 10.2105/AJPH.2016.303375 27626350 PMC5024387

[21] Resnikoff S , Lansingh VC , Washburn L , et al . Estimated number of ophthalmologists worldwide (international Council of ophthalmology update): will we meet the needs? Br J Ophthalmol 2020;104:588–92. 10.1136/bjophthalmol-2019-314336 31266774 PMC7147181

[22] Palmer JJ , Chinanayi F , Gilbert A , et al . Mapping human resources for eye health in 21 countries of sub-Saharan Africa: current progress towards vision 2020. Hum Resour Health 2014;12:44. 10.1186/1478-4491-12-44 25128163 PMC4237800

[23] Ebener S , Stenberg K , Brun M , et al . Proposing standardised geographical indicators of physical access to emergency obstetric and newborn care in low-income and middle-income countries. BMJ Glob Health 2019;4:e000778. 10.1136/bmjgh-2018-000778 PMC6623986 31354979

[24] Zhang JH , Ramke J , Mwangi N , et al . Global eye health and the sustainable development goals: protocol for a scoping review. BMJ Open 2020;10:e035789. 10.1136/bmjopen-2019-035789 PMC7202701 32193274

[25] World Health Organization . Eye care service assessment tool, 2015. Available: https://www.who.int/blindness/publications/ECSAT_EN.pdf?ua=1 [Accessed 09 Nov 2020].

