# Supplementary material for: Eye health indicators for universal health coverage: results of a global expert prioritisation process
Source: Br J Ophthalmol. 2021 Mar 12;106(7):893–901. doi: 10.1136/bjophthalmol-2020-318481 (PMC9234411; doi:10.1136/bjophthalmol-2020-318481)
Supplement: Supplementary data [file bjophthalmol-2020-318481supp001.pdf]

Appendix 1. Initial long-list of 200 eye health indicators

Inputs and Processes

Governance

Eye health is integrated into the national health plan (Y/N)
Is eye health integrated into the plans, policies and budget of other initiatives such as maternal and child care, diabetes care, school health programs, healthy aging (Y/N)
Does the country have a national eye care coordinator/focal person for eye health? (Y/N)
Does the country have a National Prevention of Blindness/Eye Health committee? (Y/N)
Is there a National Prevention of Blindness/Eye Health plan (endorsed by the Ministry of Health?) (Y/N)
If yes, is the National Prevention of Blindness/Eye Health plan implemented at country level? (Y/N)
If yes, does the National Prevention of Blindness/Eye Health plan include a budget? (Y/N)
If yes, is the National Prevention of Blindness/Eye Health plan based on evidence of eye health need gathered in the last ten years (quality of plan)
Ability to influence policy: Number of degrees of reporting separation from national eye health officer and key decision makers / Ministers and Permanent Secraries or equivalent
Number of meetings of the Prevention of Blindness/Eye Health committee, led by Ministry of Health or with the participation of MoH representative, and held in the previous year
The number of national (or sub-national) eye care status and monitoring reports developed over the previous 2 years (and proportion of planned reported actually completed)
National eye health policies, plans and programmes refer to a multisectoral approach (Y/N)
Are there any eye health programs being led and implemented by other sectors such as education or labor? (Y/N)
Eye health is incorporated into relevant poverty-reduction strategies, initiatives and wider socioeconomic policies (Y/N)
National health plan includes human resources for eye care (Y/N)
Do stakeholder groups include representatives from disabled people's organisations (DPOs) or specific disease associations (e.g. diabetes associations)? (Y/N)
Is there eye health service user (e.g. disabled people's organisations (DPOs) or disease-specific patient groups) engagement in governance? (Y/N)
Is there a regulatory framework for the privatization, commercialization and marketization of eye health services? (Y/N)
Is there legislation that makes specific eye care services such as screening for retinopathy of prematurity mandatory (Y/N)

Financing

Eye health is integrated into the national health budget (Y/N)
Proportion of population covered with health insurance (with breakdown by type of insurance)
Proportion of population covered with health insurance that includes eye care (with breakdown by type of insurance)
The percentage of defined eye health services (e.g. cataract surgery, glaucoma meds, DR laser, antiVEGF) covered by national health insurance
Total (public and private) national eye health expenditure as the percentage of total (public and private) national health expenditure

Public (government) spending on eye health as % of total health expenditure
Publically funded spending (as taxes, social contributions, compulsory private insurance contributions or other government revenues) on eye health as a percentage of current eye health expenditure
Externally funded spending (financing flows from external sources that provide the funds to public and private financing schemes) on eye health as a percentage of current eye health expenditure
Central and local council budget allocations for eye health in decentralised systems
Percent of government health budget spent on outpatient vs inpatient eye care
Is user fee revenue generated from eye care ring-fenced for eye health at facility and district level? (Y/N)
Proportion of annual national expenditure on eye care medicines by government budget, donors, charities and private patients
Share of population eligible for a defined set of eye health care goods and services funded under public programmes.
Proportion of eye care services delivered with user fee exemptions and/or waivers to access
**Infrastructure**
Number of tertiary (specialised/teaching/no onward referral) ophthalmology centres per million population
Number of secondary (district level) ophthalmology/eye care centres per million population
Number of facilities providing refraction & dispense spectacles (by private & public) per million population
Number of primary care facilities with eye health capacity (optometrists/ ophthalmic nurses/health workers trained in eye care stationed at facility) in health system per million population
Is there an active national programme for descentralisation of eye care services (Y/N)
The number and proportion of hospital beds allocated to eye care nationally
Number of tertiary centres providing specialised child eye care per million population
Number (and percentage) of neonatal units providing screening for retinopathy of prematurity nationally
Number of centres for medical management of retinal causes of vision loss (including DR) per million population
Number of centres for surgical management of retinal causes of vision loss (including DR) per million population
Number of centres providing laser treatment for diabetic retinopathy per million population
Number of centres providing glaucoma surgery per million population
Number of centres which have a functioning visual field perimeter for glaucoma services
At national level, proportion of health administrative areas with permanent (i.e. staffed full-time) eye-care services
At national level, proportion of health administrative areas with cataract surgical services
Number of low vision centres nationally (secondary and tertiary) per million population
Proportion of secondary and tertiary level centres with trained and functional units for equipment maintenance
Numbers of equipment training programmes and numbers of technician personnel trained
**Eye health workforce**

Ophthalmologist density and distribution (per million population and by urban/rural or districts), by age and sex
The proportion of ophthalmologists (as described by distribution and density) actively performing eye surgery (in the past year)
Optometrists are recognised (and regulated) by authorities and governmental structure (Y/N)
Optometrist density and distribution (per million population and by urban/rural or districts), by age and sex
Allied ophthalmic personnel (per million population and by urban/rural or districts), by age and sex
Cataract surgeons (non-ophthalmologist) are recognised by relevant government departments? (Y/N)
Number of cataract surgeons (non-ophthalmologist) (per million population and by urban/rural or districts), by age and sex
Population served by one ophthalmologist/ one cataract surgeon (rural/ urban)
Trends in eye care workers (by public and private sector) over the past 5 years; percentage increase/decrease in number per million population, by cadre
Ratio of urban to rural eye health workers per million population, by cadre
Are there incentives for ophthalmologists to work in poor or rural areas? (Y/N)
Ratio of private to public sector eye health workers, by cadre
Number of paediatric ophthalmologists and other sub-specialty trained providers (such as retina, cornea, glaucoma and low vision) per population (ratio)
Median remuneration of eye health workers, by cadre
Foreign-trained eye health workers as a percentage of total, by cadre
Number of graduates from eye health education and training programmes in the past academic year per million population, by age and sex
Number of training positions available (by cadre) that include clinical exposure of trainees or internship (e.g. ophthalmologists, refractionists/optometrists, nurses)
Techniques taught in residency programs are responsive to available infrastructure (Phaco - SICS - ECCE) (Y/N)
Is Primary Eye Care integrated into the national Primary Health Care programme? (Y/N)
Is Primary Eye Care integrated into the national Primary Health Care training? (Y/N)
Does the national programme for Community Health Workers include eye health? (Y/N)
Does the human resources for health strategic plan include eye care? (Y/N)
Do human resources for health collection systems include information on eye care staff? (Y/N)
Are human resources for health policies relevant to eye care staff and followed by eye care providers? (Y/N)
Proportion of secondary and tertiary eye care centres with trained eye care management personnel
**Supply chain**
Total number of pharmaceuticals specifically for eye care on the National Essential Medicines List
Does national procurement data collected include information on pharmaceutical products specifically for eye care? (Y/N)
Government expenditure on pharmaceuticals (medicine and consumables) specifically for eye care (e.g. eye drops, lenses)
Private expenditure on pharmaceuticals specifically for eye care
Average availability (across facilities) of selected essential eye medicines at government eye care facilities

Average availability (across facilities) of selected affordable devices/services at government low vision eye care facilities
Median consumer price ratio of selected essential eye medicines (e.g. timolol) in public and private health facilities
Medical equipment availability (equipment to be selected)

Information

Existence of a National Health Information System that includes eye care data (Y/N)
Do health authorities' reports include information related to eye care? (Y/N)
Do disease surveillance reports received at the various levels of the health system include information on eyes? (Y/N)
Number of eye care indicators included in the monitoring framework of the national health strategic plan (or other health plans)
Is disaggregation of data by equity measures (e.g. sex, urban/rural) available in eye health reporting (Y/N)
The proportion of districts reporting eye care information (annually)
Proportion of public eye care facilities that measure performance indicators annually to implement improvement plans
The proportion of private eye care facilities reporting eye care information to national information system
Reports of health authorities at various levels of the health system include information on eye care
Number of prevalence/population-based VI/eye health surveys completed during the past five years
Number eye care service assessments completed over the last five years
Reported evidence of research on the cost– effectiveness of eye health programmes
Eye health is listed as a research priority by national research funding bodies (Y/N)
The total funds available for eye health research annually in the country
The total number of eye health research projects occurring annually in the country

Outputs

Service access and readiness

Number of patient consultations (episodes) for eye diseases in the country per year - ICD code, or other national health condition or care coding system, over a set period.
The mean/median number of outpatient consultations per ophthalmologist per year
The mean/median number of cataract surgeries per cataract surgeon per year
Absence rate (days missed per year) of eye health workers, by cadre
Number of ophthalmic outpatient visits per capita per year (by new and repeat): the number of outpatient visits, over the total population
Number of eye hospital discharges per 100,000 population: this indicator provides additional information on the availability and access to inpatient
Cataract surgical rate; including variation in rate across regions/districts
Trend in CSR over 5 years; percentage increase/decrease in number per million population
Number of refractions in government eye care facilities, per year
Number of prescribed corrections from refractions in government eye care facilities, per year

Number of distance vision spectacles dispensed in government eye care facilities, per year
Number of near vision spectacles dispensed in government eye care facilities, per year
Number of refractions in private eye care facilities, per year
Number of prescribed corrections from refractions in private eye care facilities, per year
Number of distance vision spectacles dispensed in private eye care facilities, per year
Number of near vision spectacles dispensed in private eye care facilities, per year
Number of patients receiving anti-VEGF DR treatment, per year
Number of patients receiving laser DR treatment, per year
Number of assistive products dipsensed to low vision/ rehabilitation clients
Percentage of population living within standard distance (e.g. 20 km) of eye health facility at primary level
Proportion of cataract surgery operations that take place in the private vs public sector
Proportion of refractions that take place in the private vs public sector
Utilization of private providers for eye care services in rural vs. urban areas per type of provider
Proportion of hospitalizations (or number of hospital days) that take place in the private vs. the public sector
Number of days delay in presenting for emergency care (e.g. for trauma / corneal infection / retinal detachment)

Service quality and safety

Cataract surgery outcome (visual acuity) - proportion of eyes with 'good' outcome (6/18 or better)
Intraocular lens implantation rate: Proportion of all cataract operations performed with intraocular lens implantation in the previous year
Percentage Posterior Capsule Rupture and vitreous loss, per surgeon/institution
Implementation of a pre and post-operative cataract surgical counseling program (Y/N)
Total number of clinical practice guidelines on priority eye diseases and eye care pathways endorsed by the national/state health and/or clinical
Are national policies for promoting quality of care followed by eye care providers? (Y/N)
Existence of national eye care quality standards adapted to local level
Clinical supervision by district level supervisor include eye care services
Do national therapeutic guides with standardized treatments include common eye health problems?
Is there eye health workforce regulation and formal CPD requirements, by cadre (Y/N)
Is the sale/supply of spectacles formally regulated? (Y/N)
Amount of funding for quality improvement research
Rate of hospital-acquired infections (in eye services)

Efficiency

Average waiting time (days from referral/diagnosis) for ocular cancer treatment
Average waiting time (days from referral/diagnosis) for wet AMD treatment

Average waiting time (days from referral/diagnosis) for cataract surgery
Number of wet AMD patients with assessment/treatment within national target time
Number of ocular cancer patients with assessment/treatment within national target time
Costs per hospitalisation for cataract surgery ($ per patient)
Costs per cataract case at different levels of care ($ per patient)
Cataract outreach programme adherence: proportion of people with operable cataract identified through outreach who receive cataract services
School eye health programme adherence: proportion of children with referable eye/vision condition identified through school-based programme who receive services
**Responsiveness and Patient Experiences**
Number and proportion of eye care facilities or programmes in the country that undertake a survey or questionnaire on clients' experience of care, in specified period of time
Number and propotion of eye care facilities in the country that have undertaken an audit of infrastructure accessibility
The average percentage (across facilities) of eye care appointments, treatments or follow-ups skipped due to costs
The average percentage (across facilities) of prescribed eye medicines skipped due to costs
The average percentage (across facilities) of prescribed spectacle prescriptions skipped due to costs
Price of a consultation and a cataract surgery operation compared to (national) standard of living (reported as GDP per capita)
Price of a consultation and spectacles compared to (national) standard of living (reported as GDP per capita)
Eye care professional spending enough time with patients during the consultation, by cadre
Eye care professional providing easy-to-understand explanations, by cadre
Eye care professional giving opportunity to ask questions or raise concerns, by cadre
Eye care professional involving patients in decisions about care or treatment, by cadre
Have disabled people's organisations (DPOs), specific disease associations and other groups relevant to eye care the capacity and opportunity to advocate for eye health issues? (Y/N)
Have disabled people's organisations (DPOs), specific disease associations and other groups relevant to eye care the capacity and opportunity to use, analyse and feedback to government on health sector goals, planning, budgeting, expenditure and data related to eye health (Y/N)
**Care-seeking behaviour**
Number and proportion of districts with community awareness and education programmes aimed at stimulating demand for eye care services
Number of patients who received education and awareness resources/programmes for management of glaucoma
Number of patients who received education and awareness resources/programmes for management of diabetes
**Outcomes**
**Coverage of interventions (Prevention and treatment?)**
Cataract surgical coverage & effective cataract surgical coverage

Refractive error coverage & effective refractive error coverage
Presbyopic correction coverage & effective presbyopic correction coverage
Coverage of DR screening of all people with diabetes (at the frequency recommended in national guidelines)
Coverage of retinopathy of prematurity screening of all infants who require screening (according to national guidelines)
Coverage of school eye health programmes for schools nationally
Coverage of low-vision services (consultation/devices) for persons with functional low vision
Primary eye conditions service coverage & effective primary eye conditions service coverage
The proportion of the population with a self-reported (met and) unmet need for eye care services (during the last 12 months)
In trachoma endemic countries, proportion of endemic communities covered by the SAFE strategy
In onchocerciasis endemic countries, ivermectin coverage (%) of districts at high risk of onchocerciasis
In onchocerciasis endemic countries, therapeutic ivermectin coverage rate of total population at high risk of onchocerciasis
**Equity**
eCSC/eREC for the poorest 40% of the population
Health insurance coverage for the poorest 40% of the population
Distance from clinic for poorest 40% of the population
Perception of exclusion and inclusion by the eye health system
**Impact**
**Improved eye health outcomes and equity**
Prevalence of blindness/MSVI/mild VI; by SEP, PoR, sex
Prevalence of near vision impairment; by SEP, PoR, sex
Prevalence of VI (by categories) due to avoidable causes; by SEP, PoR, sex
Prevalence of childhood vision impairment and blindness
Cause specific prevalence of vision impairment and blindness
The proportion of the population achieving 6/12 vision (UCVA or CVA)
Number (and %) of districts in the country where blinding trachoma is a public health problem
Number (and %) of districts in the country where onchocerciasis is a public health problem
**Financial (and social) risk protection**
Incidence of “catastrophic” eye health expenditures
Incidence of impoverishment due to out-of-pocket eye health payments
**Well-being / Quality of life**
Employment rate of people with vision impairment (following treatment and/or rehabiliation in eye health services, compared to general population employment rate
The proportion of the population 'mostly' or 'completely' satisified with their eye health

Change in health-related quality of life scores following eye care intervention
**Eye Health Determinants**
Population age structure: What proportion of the population is of school-going age (4-18 years)
Population age structure: What proportion of the population is aged 50 years or older
Air pollution level in cities (Annual mean levels of fine particulate matter in cities, urban population (micrograms per cubic meter)) [SDG 11.6.2]
Population with primary reliance on clean fuels and technologies [SDG 7.1.2]
Measles containing vaccine second dose (MCV2) coverage amongst children by the nationally recommended age
Rubella containing vaccine coverage amongst children by the nationally recommended age
Age-standardized prevalence of current tobacco use among persons aged 18+ years
Age-standardized prevalence of raised blood glucose/diabetes among persons aged 18+ years
Population using safely managed drinking-water services [SDG 6.1.1]
Population using safely managed sanitation services [SDG 6.2.1a/6.2.1b]
