# Supplementary material for: Eye health indicators for universal health coverage: results of a global expert prioritisation process
Source: Br J Ophthalmol. 2021 Mar 12;106(7):893–901. doi: 10.1136/bjophthalmol-2020-318481 (PMC9234411; doi:10.1136/bjophthalmol-2020-318481)
Supplement: Supplementary data [file bjophthalmol-2020-318481supp002.pdf]

Appendix 2. Additional information on study methods.

Round 1

Almost half the long-list indicators were input & process indicators (n=98), with fewer than 20 indicators in each of the outcome, impact and determinants domains.

The panel were asked to:
"Please consider each indicator - and the information it aims to synthesise - and prioritise according to its relevance in your setting, keeping in mind that the feasibility, validity, reliability and comparability of priority indicators will be scrutinised in the next round.
There are five options to select from:"

Value	Scale	Definition
0	Redundant	This option is for use only when another indicator within a domain adequately or better captures the same information
1	No need to collect	The indicator is not relevant for monitoring eye health in your setting
2	Low priority to collect	The indicator would not be particularly useful to allow effective monitoring of eye health in your setting
3	High priority to collect	The indicator would be useful to allow effective monitoring of eye health in your setting
4	Essential to collect	The indicator must be collected to ensure effective monitoring of eye health in your setting

A review of Round 1 scoring resulted in amendments to three groups of indicators:

1. Refractive error: No *refractive error*-related indictors scored in the top half of the list, though most had scored five or more '0's, where participants deemed the indicator covered by another in the list. This suggests votes were split across two or more similar options and refraction indicators were potentially underrepresented. We proposed a new *refraction rate* indicator (the refraction equivalent of the cataract surgical rate) to be added to Round 2.
2. Financial Risk Protection: To remain aligned with our UHC framework, both *financial risk protection* indicators – the incidence of catastrophic and impoverishing spending on eye health care – were retained, despite not scoring in the top half. A *health insurance coverage* indicator was maintained alongside these financial risk protection indicators despite placing just below the median score.
3. Governance: Three *governance* indicators that scored highly were duplicate concepts of ones retained.

Priority scoring was not consistent across the domains of HIS, with a higher proportion of input & process indicators featuring in the top half of the scoring compared to any other domain.

Round 2

Two panelists' core ranking responses were excluded (one not completed, one incorrectly completed). Eight responses included duplication of a rank position 1-10 resulting in more than 10 choices. These were modified such that one duplicate position was selected at random and modified as 'rank + 1' and all subsequent ranking positions following the duplication were modified as 'rank + 1', the lowest scoring choice was deleted and consecutive ranks 1 through 10 were recorded.

Appendix 3. The top 30 global eye health indicators prioritised by 72 global eye health stakeholders in Round 2

**Inputs & processes**
**Governance**
Eye health is integrated into the national health plan (or the relevant specific plan e.g. NCD plan) (Y/N)
National health plan includes human resources for eye care (Y/N)
Is the National Prevention of Blindness/Eye Health plan implemented at country level? (Y/N)
Is the National Prevention of Blindness/Eye Health plan based on evidence of eye health need gathered in the last ten years (quality of plan) [e.g. cites population-based survey and health service assessment data]
**Finance**
Eye health is integrated into the national health budget (Y/N)
The percentage of defined eye health services (e.g. cataract surgery, glaucoma meds, DR laser, antiVEGF) covered by national health insurance
**Infrastructure**
Number of primary care facilities with eye health capacity (by any of optometrists/ ophthalmic nurses/health workers trained in eye care) per million population
Number of secondary (district level) ophthalmology/eye care centres per million population
Number of low vision centres nationally (secondary and tertiary) per million population
Number (and percentage) of neonatal units providing screening for retinopathy of prematurity nationally
**Supply chain**
Total number of pharmaceuticals specifically for eye care on the National Essential Medicines List
**Information**
Existence of a National Health Information System that includes eye care data (Y/N)
Number of eye care indicators included in the monitoring framework of the national health strategic plan (or other health plans) [Reported as number and proportion of total]
Is disaggregation of data by equity measures (e.g. sex, urban/rural) available in eye health reporting (Y/N)
**Eye health workforce**
Ophthalmologist density and distribution (per million population and by urban/rural or districts), by age and sex; include 5 year trend option
Is Primary Eye Care (prevention, identification, treatment, referral as appropriate per setting) integrated into the national Primary Health Care programme? (Y/N)
Is Primary Eye Care integrated into the national Primary Health Care training? (Y/N)
Does the human resources for health strategic plan include eye care? (Y/N)
**Outputs**
**Access**
Cataract surgical rate; including variation in rate across regions/districts & 5 year trend in CSR
**Quality & safety**
Cataract surgical outcome (visual acuity) - proportion of eyes with 'good' outcome (6/18 or better)
**Outcomes**
**Coverage**
Cataract surgical coverage & effective cataract surgical coverage
Refractive error coverage & effective refractive error coverage
Coverage of school eye health programmes for schools nationally
Coverage of retinopathy of prematurity screening of all infants who require screening (according to national guidelines)
Coverage of DR screening of all people with diabetes (at the frequency recommended in national guidelines)
**Equity**
Effective cataract surgical coverage & effective refractive error coverage for the poorest 40% of the population
**Impact**
**Improved outcomes**
Prevalence of blindness/MSVI/mild VI; by SEP, PoR, sex [equity dimensions to be reported as available]
Cause specific prevalence of vision impairment and blindness; by SEP, PoR, sex [equity dimensions to be reported as available]

Prevalence of VI (by categories) due to avoidable causes; by SEP, PoR, sex [equity dimensions to be reported as available]
Prevalence of childhood vision impairment and blindness; by SEP, PoR, sex [equity dimensions to be reported as available]
